# Transcriptional regulation and its misregulation in Alzheimer’s disease

**DOI:** 10.1186/1756-6606-6-44

**Published:** 2013-10-21

**Authors:** Xiao-Fen Chen, Yun-wu Zhang, Huaxi Xu, Guojun Bu

**Affiliations:** 1 Fujian Provincial Key Laboratory of Neurodegenerative Disease and Aging Research, Institute of Neuroscience, College of Medicine, Xiamen University, 361102 Xiamen, Fujian, People’s Republic of China

**Keywords:** Alzheimer’s disease, Transcription factors, Transcriptional regulatory element, Polymorphism, Amyloid-β

## Abstract

Alzheimer’s disease (AD) is a devastating neurodegenerative disorder characterized by loss of memory and cognitive function. A key neuropathological event in AD is the accumulation of amyloid-β (Aβ) peptide. The production and clearance of Aβ in the brain are regulated by a large group of genes. The expression levels of these genes must be fine-tuned in the brain to keep Aβ at a balanced amount under physiological condition. Misregulation of AD genes has been found to either increase AD risk or accelerate the disease progression. In recent years, important progress has been made in uncovering the regulatory elements and transcriptional factors that guide the expression of these genes. In this review, we describe the mechanisms of transcriptional regulation for the known AD genes and the misregualtion that leads to AD susceptibility.

## Introduction

Alzheimer’s disease (AD) is an age-associated neurodegenerative disease and is the most common form of dementia in the elderly. Like many other geriatric disorders, AD appears to be multifactorial in its origin. Mounting evidence from genetic, pathological, and functional studies has shown accumulation of amyloid-β (Aβ) peptide in the aging brain [1,2]. Aβ aggregates in the forms of soluble Aβ oligomers and amyloid plaques trigger numerous pathophysiological changes that ultimately lead to cognitive dysfunction [3-5]. Aβ is a 40–42 amino-acid peptide that is generated through multiple proteolytic cleavages of the amyloid-β protein precursor (APP) [6]. The 'amyloid hypothesis’ postulates Aβ as the common initiating factor in AD pathogenesis and thus places Aβ as the hot research focus in the past two decades [2]. Emerging evidences have indicated that an imbalance between production and clearance of Aβ in the brain leads to AD pathogenesis [3]. A large group of genes have been described to affect Aβ generation or clearance, which are part of the 'AD genes’.

Although it is clear that expression levels of AD genes are important in AD etiology, much remains unknown about their specific regulation [7]. Studying the regulatory elements of disease genes and their corresponding transcription factors is therefore critically important for elucidation of the disease processes [8]. This review will discuss the mechanisms of transcriptional regulation for AD genes, and the misregulation that leads to AD susceptibility.

### Transcription regulation of *BACE1*

Aβ is derived from sequential cleavage of APP by β- and γ-secretase [9]. The initiation of Aβ production by BACE1 and the disease-associated increase of BACE1 level places BACE1 in the central role of AD pathogenesis [10-13]. Numerous efforts have been devoted to inhibiting BACE1 expression and activity to reduce Aβ production and its associated neuronal toxicity [14]. BACE1 is an aspartyl protease which cleaves APP at the known β-secretase sites of Asp + 1 and Glu + 11 of Aβ [15]. *BACE1* knockout mice do not produce Aβ and are free from AD-associated pathologies including memory deficits and neuronal loss [16,17]. However, detailed studies revealed specific behavioral and physiological alterations in the complete absence of BACE1 [18-20]. It was suggested that non-APP substrates that are subjected to BACE1 cleavage might be important for these specific behavioral and functional changes in *BACE1*-deficient mice [9].

The *BACE1* gene spans about 30 kilobases (kb) on chromosome 11q23.2 and includes nine exons [14]. Ever since it was first cloned in 2003, the *BACE1* gene promoter has attracted extensive studies [14,21]. This promoter lacks the typical CAAT and TATA boxes but has a very high GC content at its proximal region [21]. The first 600 bp of the promoter is highly conserved amongst rat, mouse and human, suggesting that this region contains important regulatory elements which modulate *BACE1* transcriptional activity [21]. A large amount of evidence shows that the *BACE1* promoter contains multiple transcription factor-binding sites and is typical of an inducible expression [14,21,22]. A number of transcription factors have been suggested to control *BACE1* transcription, including specificity protein 1 (SP1), NF-κB, hypoxia inducible factor 1α (HIF-1α), and peroxisome proliferator-activated receptor-gamma (PPAR γ), amongst others.

Sp1 belongs to the Sp/KLF (Specificity protein/Krüppel-like factor) family and is amongst the first transcription factor identified to regulate *BACE1* gene expression [23,24]. Deletion analysis of *BACE1* promoter and the gel shifting assay demonstrated the functional binding site for Sp1 on the *BACE1* promoter. Subsequently, Sp1 over-expression potentiated the activity of the wild-type, but not of the Sp1-binding-site-mutant *BACE1* promoter, demonstrating an activator function for Sp1 in *BACE1* expression. Furthermore, the lack of endogenous Sp1 protein in Sp1-knockout cells markedly reduces *BACE1* promoter activity. These results clearly show that Sp1 modulates the endogenous *BACE1* expression [24]. The crucial role of Sp1 in regulation of *BACE1* expression was supported by different experimental approaches. Mithramycin A, which inhibits Sp1 binding to DNA, reduced *BACE1* expression in a dose-dependent manner [24,25]. 12/15-Lipoxygenase (12/15-LO), an enzyme widely distributed in the central nervous system, elevated the levels of *BACE1* mRNA and protein through a Sp1-mediated transcription control [26]. Considering the activation role of Sp1 for *BACE1* expression, future studies are need to illustrate the spatial and temporal expression patterns, and the transcriptional activity of Sp1 in distinct cell types of the brain. Importantly, Sp1 is known to interact with NF-kB which also regulates *BACE1* expression level, it remains to be determined whether they regulate *BACE1* gene expression in a synergistic manner [27,28].

NF-κB is a unique transcription factor that regulates *BACE1* transcription in a cell type-specific manner [29]. A detailed analysis using *BACE1* promoter constructs revealed that NF-κB acts as a repressor for *BACE1* transcription in differentiated neuronal cultures and non-activated glial cultures, but as an activator for *BACE1* transcription in activated astrocytic and Aβ-exposed neuronal cultures. The effects of NF-κB on the regulation of *BACE1* transcription are mediated by the binding of distinct NF-κB subunits. The p50/c-Rel heterodimer acts as repressor, while p50/p65, p52/c-Rel or p52/p65 acts as activator when binding to *BACE1* promoter-specific NF-κB site. Recently, it was found that NF-κB differently regulates Aβ production under physiological and supraphysiological Aβ concentrations by modulating secretase expression [30]. Under physiological conditions, NF-κB lowers the transcriptional activity of *BACE1* promoter and triggers a repressive effect on Aβ production. However, NF-κB activates the transcription of *BACE1* promoter and enhances Aβ production under pathological context. Thus, using compounds to modulate *BACE1* expression based on NF-κB might lead to different outcomes under different conditions.

HIF-1 is a hetero-dimeric transcription factor composed of an oxygen-regulated alpha-subunit (HIF1α) and a constitutively expressed and stable beta-subunit (HIF1β) [31]. Under hypoxic conditions, HIF-1 binds to a hypoxia-responsive element (HRE) on a target gene promoter and activates gene expression [32]. A functional HRE was identified in human and mouse *BACE1* gene promoter [33,34]. Indeed, hypoxia augments β-secretase cleavage of APP by increasing *BACE1* gene transcription both in vivo and in vitro. The effect of hypoxia on *BACE1* expression is presumably mediated by HIF-1α. Over-expression of HIF-1α increased *BACE1* mRNA and protein levels, whereas down-regulation of HIF-1α reduced the level of *BACE1* expression. Consistent with these results, *BACE1* expression was reduced in the hippocampus and the cortex of HIF-1α conditional knock-out mice [34]. Additionally, hypoxia treatment markedly increased Aβ deposition and neuritic plaque formation and potentiated the memory deficit in Swedish mutant APP transgenic mice [33]. Recently, it was shown that hypoxia up-regulates *BACE1* expression through two distinct mechanisms: an early release of reactive oxygen species from mitochondria and a late activation of HIF-1α [35]. Interestingly, salidroside, which has long been used in traditional Tibetan medicine to relieve high altitude sickness, is able to attenuate Aβ accumulation via HIF-1α-mediated reduction of *BACE1* expression [36]. The link between hypoxia and *BACE1* expression provides a molecular mechanism for increased incidence of AD following cerebral ischemic and stroke injuries.

Peroxisome proliferator-activated receptor-gamma (PPARγ) is a ligand-activated nuclear transcription factor that has two isoforms, PPARγ1 and PPARγ2 [37,38]. These isoforms are produced by alternative splicing of the same gene. PPARγ can form heterodimers with retinoid X receptors (RXR) and binds to PPAR-responsive element (PPRE) upon ligand activation [39]. The *BACE1* gene promoter also contains PPRE and mutagenesis of the PPRE increased *BACE1* gene promoter activity by abolishing PPARγ binding to PPRE [40]. Over-expression of PPARγ has been shown to reduce *BACE1* gene promoter activity. These results suggest a repressive role of PPARγ on *BACE1* expression. Interestingly, brain extracts from AD patients showed that both PPARγ levels and binding to PPRE on the *BACE1* gene promoter was decreased [40]. Pro-inflammatory cytokines decrease PPARγ mRNA level and this effect was suppressed by non-steroidal anti-inflammatory drugs (NSAIDs). Intriguingly, NSAIDs were shown to modulate *BACE1* transcription by repressing its promoter activity specifically through PPARγ [40]. Indeed, epidemiological evidence suggests that a strong inflammatory reaction is present in AD brains and long-term treatments with NSAIDs decrease the risk for AD [41]. PPARγ could be herein a major regulatory factor for modulating inflammation. The activation of PPARγ by agonists such as certain NSAIDs could open a prospective avenue for AD therapy.

### Transcription regulation of *APOE*

Apolipoprotein E (ApoE) is a major cholesterol carrier in the brain [42,43]. ApoE is primarily produced by astrocytes and its function is to deliver lipids to neurons through the binding of cell surface ApoE receptors [44]. Human ApoE exists as three polymorphic alleles: ϵ2, ϵ3 and ϵ4 [43]. These three isoforms differ from each other by a single amino acid, resulting in different protein structures, lipid association and receptor binding [45-47]. The ϵ4 allele of the ApoE is the strongest genetic risk factor for late-onset AD (LOAD) [48]. Individuals with one ϵ4 allele are 3–4 times more likely to develop AD than those without ϵ4 allele [49]. Interestingly, the rare ϵ2 allele has a protective effect against AD compared with the ϵ3 allele [50].

In addition to the polymorphisms at the ϵ2/ϵ3/ϵ4 locus, changes in *APOE* expression level have been reported to be associated with AD, although the results remain controversial [51]. ApoE levels have been found to be increased in the cerebrospinal fluid (CSF), plasma and frontal cortex of AD patients [52-54]. However, other studies have observed either no change or a decrease in the ApoE levels of AD patients [55-58]. Such discrepancies may be related to confounding factors interfering with sample handling and/or analyses, of which remains to be clarified. Indeed, one study pinpointed that the hydrophobic character of ApoE resulted in adsorption to different types of test tubes commonly used for collection of CSF at lumbar puncture, resulting in falsely low levels [58]. More recently, two groups showed consistent results that reducing human ApoE level attenuates amyloid deposition in mutant human APP transgenic mouse model, regardless of isoform status [59,60]. Thus, overall *APOE* expression level plays an important role in AD pathology, although the exact correlation remains controversial.

*APOE* expression is regulated by nutritional, developmental and hormonal factors which bind to its proximal promoter region [61-63]. In contrast to *BACE1*, the 5’-flanking sequence of *APOE* harbors a functional TATA box [64]. Multiple cis-acting positive and negative regulatory elements have been mapped to the 5'-flanking sequence of *APOE*, including AP-2, PPARγ and liver X receptor (LXR) [65-68].

AP-2 is an astrocyte-associated transcription factor whose expression can be strongly and rapidly induced by cyclic AMP (cAMP) [69]. Retinoic acid (RA) is also known to regulate the transcriptional activity of *AP-2* gene [70]. Interestingly, the activity of the proximal *APOE* promoter in astrocytes is up-regulated by cAMP and RA synergistically [65]. Sequence analysis and foot-printing technique revealed the existence of two binding sites for AP-2 in the *APOE* promoter which might mediate the stimulatory effect of cAMP and RA [65]. Mutations in these regions markedly impaired the trans-stimulatory effect of AP-2 on *APOE* expression [65]. These results indicate the existence of functional AP-2 sites in the promoter region of ApoE. The AP-2 transcription factor family consists of five isoforms (α, β, γ, δ and ϵ), with α- and β-isoform abundantly expressed in the brain [71,72]. Interestingly, a recent study observed that Aβ induced a time-dependent increase in *APOE* mRNA in astrocytes which was mediated by AP-2β [73]. The transcriptional up-regulation of *APOE* level by Aβ may be a neuro-protective response against Aβ-induced cytotoxicity, consistent with ApoE’s role in cytoprotection.

Proliferator-activated receptor gamma (PPARγ) and liver X receptors (LXRs) form obligate hetero-dimers with retinoid X receptors (RXRs) and are reported to regulate *APOE* transcription [66,68]. Indeed, the LXR agonists GW3965 and TO901317 were reported to increase *APOE* expression in astrocytes, enhance Aβ clearance and ameliorate the memory deficit in amyloid mouse model [66,67]. Similar to LXRs, PPARγ agonists such as pioglitazone and ciglitazone can also induce *APOE* expression and rescue the behavioral deficits in AD mouse model [39,68]. In addition, RXR activation by numerous compounds has shown to increase *APOE* level, likely through activation of RXR and PPAR signaling pathways [74,75]. Owing to their ability to enhance *APOE* gene expression and promote Aβ degradation, LXRs, PPARs, and perhaps RXRs, serve as an attractive therapeutic target for AD.

While rarely-detected on the *BACE1* gene promoter, polymorphisms within the proximal promoter of the *APOE* gene lead to changes in ApoE level by altering gene transcription [76]. Four promoter polymorphisms have been identified and their association with AD risk has been investigated, including -491 (A/T transversion), -427 (T/C transition), -219 (G/T transversion, also known as the Th1/E47cs polymorphism), +113 (C/G transversion, also termed IE1) [77-80]. These polymorphisms are proposed to affect the transcriptional activity of ApoE gene by altering the binding of transcription factors [81]. Among them, the -491 A/T polymorphism has been the most thoroughly investigated and shown to robustly affect ApoE level. The A to T substitution at -491, and the T to G substitution at -219, resulted in a 63% decrease and a 169% increase of the *APOE* promoter activity, respectively [81]. Epidemiological studies have shown that the -491 T allele was associated with a decreased risk for AD, while the -219 T allele was associated with an increased risk for AD occurrence, independently of the ϵ2/ϵ3/ϵ4 polymorphism [82]. These data suggest that these promoter polymorphisms are functional in nature. In addition to the polymorphism within the coding region, uncovering the polymorphism within the *APOE* promoter might be also beneficial to predict AD risk.

### Transcription regulation of other AD genes

APP belongs to the type I transmembrane proteins, encompassing a long extracellular domain, a hydrophobic transmembrane domain, and a short C-terminal intracellular domain [6]. The human *APP* gene is located on the long arm of chromosome 21 and contains at least 18 exons [83]. APP is abundantly expressed in the neuronal cells of the central nervous system; and the mechanisms controlling *APP* gene expression have been extensively studied [8,84-88]. The *APP* promoter is devoid of typical TATA and CAAT boxes, but contains a strong initiator element surrounding the major transcription start site [89]. The promoter sequences of *APP* gene are highly conserved among species, and share numerous binding sites for regulatory transcription factors [85,86,90,91]. The *APP* promoter activation is mainly governed by two GC-rich elements, the -93/-82 fragment (APBβ) which is bound by CCCTC-binding factor (CTCF) and the -65/-41 fragment (APBα) which is bound by stimulating protein 1 (SP1) and the upstream stimulatory factor (USF) [92-96]. Further, numerous stress factors could activate *APP* transcription which is mediated by heat-shock factor 1 (HSF-1) binding to the heat-shock element (HSE) at position -317 [97]. Another transcription factor NF-κB was found to specifically recognize two identical sequences at -2250/-2241 and -1837/-1822 on *APP* promoter. In neural cells that were treated with either the inflammatory cytokine interleukin-1beta (IL-1β) or the excitatory amino-acid glutamate, NF-κB up-regulated the transcriptional activity of the *APP* promoter [98,99]. Rac1, a member of the Rho family GTPases, was shown to stimulate the transcription of *APP* promoter in the region between -233 and -41 bp [100]. In primary hippocampal neurons, over-expression of the dominant-negative Rac1 mutant or the presence of Rac1 inhibitors decreased the levels of *APP* mRNA, indicting Rac1 could be a potential drug target for AD therapy [100]. Other regulatory elements includes the binding sites for activator protein 1 (AP1), cAMP-responsive element-binding protein (CREB) and 'GATA’ binding factor 1 (GATA1) [101]. Interestingly, copper depletion significantly reduced *APP* gene expression by acting on the region between -490 and +104 of *APP* promoter [102]. In addition, promoter polymorphisms have been found to modulate APP expression and therefore increase susceptibility to AD, including -877 T/C, -955A/G [103].

Presenilin genes (*PSEN1* and *PSEN2*) encode highly homologous integral membrane proteins which are the catalytic subunits of γ-secretase [104-106]. *PSEN* mutations cause abnormal processing of APP and lead to early onset AD [107-109]. Therefore, *PSEN* gene regulation may play a crucial role in the development of AD. Both *PSEN*s are expressed primarily in neurons [110,111]. Their promoters lack a TATA box but contain transcriptionally active GC boxes [112,113]. To date, most studies are focused on the transcriptional regulation of *PSEN1*; little is known about the transcriptional control of *PSEN2*. Deletion mapping of the human *PSEN1* promoter delineated the most active region between -22 and -6 which controls over 90% of *PSEN1* promoter activity [114]. Ets transcription factors bind to this region and activate *PSEN1* transcription [115]. Intriguingly, co-activator p300 appears to interact with Ets transcription factors and co-activate *PSEN1* transcription [115]. Zinc finger protein (ZNF237) and chromodomain helicase DNA-binding protein (CHD3) interact with Ets transcription factor ERM and inhibit *PSEN1* transcription [116,117]. Since p300 has intrinsic histone acetyl-transferase (HAT) activity and CHD3 is a component of the histone deacetylase (HDACs) complex, chromatin modification by acetylation and deacetylation may play a critical role for *PSEN1* transcription regulation [118]. In a separate study, cAMP-responsive element-binding protein (CREB) was shown to bind *PSEN1* promoter upon stimulation by N-Methyl-D-aspartate *(*NMDA) or brain-derived neurotrophic factor (BDNF), and enhance *PSEN1* transcription [119]. Further, IL-1β and Aβ42 peptide synergistically activated *PSEN1* gene expression and the effect could be enhanced by hypoxia. At least two promoter polymorphisms (-22C/T, -48C/T) have been found to modulate *PSEN1* expression and AD risk [120,121]. On the *PSEN2* promoter, a functional nerve growth factor (NGF) binds to its responsive element and leads to two-fold up-regulation of *PSEN2* transcription [122]. Early growth response gene-1 (Egr-1) binds to *PSEN2* promoter, and *PSEN2* level is increased three-fold by over-expression of Egr-1, or by 12-O-tetradecanoylphorbol-13-acetate (TPA) which increases Egr-1 level [123].

Recently, studies from two independent groups of researchers suggested that rare variants in the *TREM2* (triggering receptor expressed on myeloid cells 2) gene are associated with an increased risk of late-onset AD [124,125]. *TREM2* encodes a single-pass type I membrane receptor that regulates cell activity through a transmembrane signaling adapter protein called TYROBP (also called DAP12) [126]. In the brain, *TREM2* is dominantly expressed in microglia and performs two important roles: suppresses inflammatory reactivity and mediates the phagocytosis of cell debris [127,128]. Impaired function of the *TREM2* gene may therefore affect the inflammatory processes and the clearance of amyloid plaques, ultimately leading to increased risk for AD. Interestingly, *TREM2* expression in microglia was reduced more than 8-fold after Aβ treatment [129], which indicates that increasing *TREM2* level might be beneficial for AD therapy. Regulation of *TREM2* transcription especially in microglia remains largely unknown. Identifying the transcriptional regulators for *TREM2* expression may therefore open a new avenue for AD therapy.

## Conclusions

This present review summarizes the mechanisms of transcriptional regulation for several important AD genes and their misregulation that leads to AD susceptibility. Mounting evidence has emerged to support an important role of transcription regulation in the initiation and progression of AD. With a more thorough understanding of the changes for the gene expression profile, reciprocal drug targets can be developed to reverse the changes in transcription and alleviate AD symptoms. In addition, an alteration in gene expression presumably occurs in the early stage of the disease and accounts for the appearance of pathological hallmarks. Therefore, diagnostic techniques based on gene expression changes have the potential to detect the onset of AD before it is histologically obvious, thus allowing early treatment to prevent disease onset and provide long-lasting efficacy after discontinuation of the treatment [130].

AD therapy based upon the modulation of gene expression profiles relies heavily on a comprehensive understanding of the regulatory transcription factors and their responding elements on the promoter of AD genes. In recent years, important progress has been made in understanding the transcription regulation of *BACE1*, *APOE, APP* and *PSEN* promoters. The regulation of *BACE1* promoter activity has been extensively studied and the derived knowledge has been guiding the identification of compounds to inhibit *BACE1* expression through comprehensive drug screening. Regulation of *APOE* transcription is only partially investigated in the central nervous system and could be extremely complex. Human ApoE exists as three polymorphic alleles: ϵ2, ϵ3 and ϵ4, special attention needs to be drawn to mechanisms of differential expression of the different ApoE isoforms. Aβ peptide generation depends largely on the amount of APP substrate. Therefore, the regulation of *APP* transcription plays an important role in AD susceptibility. Several studies have observed an increase of *APP* mRNA levels in AD brains which exacerbates Aβ deposition [8,131]. The up-regulated levels of APP could be attributed to the altered binding of transcription factors to their specific positive and negative cis-elements. Because presenilins are the catalytic subunits of γ-secretase, drugs developed to inhibit the transcription of *PSEN*s could potentially reduce Aβ generation. However, presenilins cleave a large number of trans-membrane targets (such as Notch), significant side effects could be induced by down-regulating *PSEN* transcription or enzymatic activity [132-134]. Better understanding of the transcriptional properties of *PSEN*s in the future could provide a mechanistic target to potentially alleviate AD pathology; with minimal side effects. Intriguingly, a recent study demonstrated that PSEN2, but not PSEN1, plays an important role in mediating Notch cleavage [135]. PSEN2-sparing γ-secretase inhibition was suggested to a novel and efficacious γ-secretase targeting strategy for AD. Therefore, transcription factors that specifically inhibit the expression of *PSEN1*, but not *PSEN2*, would be an effective and novel drug target for AD therapy. At this present time, researchers have also focused on polymorphisms within the AD gene promoter, since single-nucleotide changes have been documented to affect transcriptional activity of AD genes. These polymorphisms may affect transcription factor binding either by directly altering a transcription factor binding site, or by changing the structure of DNA thereby affecting the access of transcription factor to the binding site.

Recently, genome-wide expression studies have been performed to investigate the complex pathogenesis of AD by using transgenic AD animals, patient-derived cell lines, and post-mortem brain tissues [136]. Changes in the transcription levels of a group of genes have been identified, although the results have been discordant, and may be possibly due to different experimental approaches used [136]. With the development of array technologies especially the RNA-seq technique, more comprehensive and accurate transcriptome analysis could be derived to interpret the pathogenesis of AD. With the increasing number of AD genes being discovered, further analysis of the transcriptional regulation of these AD genes and the variants in their regulatory regions will not only help to elucidate AD etiology, but also guide targeted drug development for AD therapy.

## Competing interests

The authors declared that they have no competing interests.

## Authors’ contributions

All authors participated in developing and discussing the ideas, integrating the information, and writing the manuscript. All authors have read and approved the final manuscript.
